# Design and technical construction of a tactile display for sensory feedback in a hand prosthesis system

**DOI:** 10.1186/1475-925X-9-50

**Published:** 2010-09-14

**Authors:** Christian Antfolk, Christian Balkenius, Göran Lundborg, Birgitta Rosén, Fredrik Sebelius

**Affiliations:** 1 Department of Electrical Measurements, Lund University, Lund, Sweden; 2 Lund University Cognitive Science, Lund, Sweden; 3 Department of Hand Surgery, Malmö University Hospital, Malmö, Sweden

## Abstract

**Background:**

The users of today's commercial prosthetic hands are not given any conscious sensory feedback. To overcome this deficiency in prosthetic hands we have recently proposed a sensory feedback system utilising a "tactile display" on the remaining amputation residual limb acting as man-machine interface. Our system uses the recorded pressure in a hand prosthesis and feeds back this pressure onto the forearm skin. Here we describe the design and technical solution of the sensory feedback system aimed at hand prostheses for trans-radial/humeral amputees. Critical parameters for the sensory feedback system were investigated.

**Methods:**

A sensory feedback system consisting of five actuators, control electronics and a test application running on a computer has been designed and built. Firstly, we investigate which force levels were applied to the forearm skin of the user while operating the sensory feedback system. Secondly, we study if the proposed system could be used together with a myoelectric control system. The displacement of the skin caused by the sensory feedback system would generate artefacts in the recorded myoelectric signals. Accordingly, EMG recordings were performed and an analysis of the these are included. The sensory feedback system was also preliminarily evaluated in a laboratory setting on two healthy non-amputated test subjects with a computer generating the stimuli, with regards to spatial resolution and force discrimination.

**Results:**

We showed that the sensory feedback system generated approximately proportional force to the angle of control. The system can be used together with a myoelectric system as the artefacts, generated by the actuators, were easily removed using a simple filter. Furthermore, the application of the system on two test subjects showed that they were able to discriminate tactile sensation with regards to spatial resolution and level of force.

**Conclusions:**

The results of these initial experiments in non-amputees indicate that the proposed tactile display, in its simple form, can be used to relocate tactile input from an artificial hand to the forearm and that the system can coexist with a myoelectric control systems. The proposed system may be a valuable addition to users of myoelectric prosthesis providing conscious sensory feedback during manipulation of objects.

## Background

In the intact hand, sensory feedback results from the stimulation and activation of the mechanoreceptors of the skin [1] and is important for the control of the hand[2].

When the signals from these receptors result from an active exploratory procedure, they can be used to perceive the shape of a novel object [3,4]. This information is also merged with contextual information and expectations based on previous experiences [5] and works together with working memory to form representations of the shape of an object [6]. In addition, the receptors of the hand and the sensory inflow are necessary to produce the feeling that the hand is a part of the body. The lack of sensation from an extremity can even give rise to effects where the extremity is no longer felt as a part of the body [7].

Today's commercial prosthetic hands provide no conscious sensory feedback to the user. To overcome this deficiency in prosthetic hands a sensory feedback system utilising a "tactile display" on the remaining residual limb acting as man-machine interface could be used. Earlier proposed systems have focused on vibrations or an electric current to convey the sensory feedback [8-11]. These systems have enhanced the ability of the user to discriminate the applied pressure/force to the prosthetic hand, but they are based on transforming one physical stimuli to another, e.g. pressure to vibration. Other systems using direct pressure to pressure feedback [12-14] using a single site of stimulation have focused on the feedback of the total grasp force in a prosthetic hand.

Targeted reinnervation surgery is the base for an elegant type of indirect sensory feedback that recently has been proposed for full-arm amputees. Targeted reinnervation takes nerves that once served the hand, a skin region of high functional importance, and redirects them to less functionally relevant skin areas, typically on the chest adjacent to the amputation site. When some of these individuals are touched on this reinnervated skin they feel as though they are being touched on their missing limb [15,16]. The proposed sensory feedback system could well be used on these patients.

For an amputee using a prosthetic hand, the absence of tactile feedback is noticeable and might influence intuitive use of the prosthesis as well as sense of ownership of the prosthesis [17-21]. There exist myoelectric prostheses with sensors that provide feedback to a closed control system which tightens a grip as slippage of an held object is detected [22]. However, the sensation is not fed back to the user and prostheses that lack the ability of giving sensory feedback to the user have a higher risk of not being used [23].

We have recently presented a new concept and initial evaluation in non-amputees of a tactile display aimed for sensory feedback in hand prostheses [24]. Our concept is based on tactile sensors in the prosthesis connected to an array of tactile stimulators on the residual limb. When the sensors are activated by touch and manipulation of objects in every day use of the prosthesis it causes a tactile stimulation on the residual limb. A system has been designed and built consisting of five actuators, control electronics and a test application running on a computer. Five actuators are mounted on the forearm skin providing sensory information to the user. Each finger is represented by one actuator providing spatial and force sensory feedback on the forearm. The concept utilises the neural mechanoreceptors for pressure in the forearm skin, hereby inducing physiologically natural tactile stimuli. The hypothesis is that the sensation produced on the forearm provides a proportional pressure sensation to the stimuli and that it can be used in coexistence with a myoelectric control system thus providing useful and acceptable feedback. Here we present the design and technical solution of such a "tactile display" together with an investigation of critical parameters.

## Methods

### Design and technical description of the system

**Figure 1:**
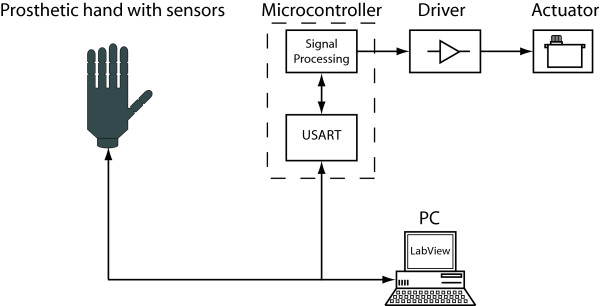
**Tactile Display and Prosthetic Hand Block Diagram**. The sensory feedback system was evaluated in a lab setting with a PC generating the stimuli wrt spatial resolution and force discrimination.

**Figure 2:**
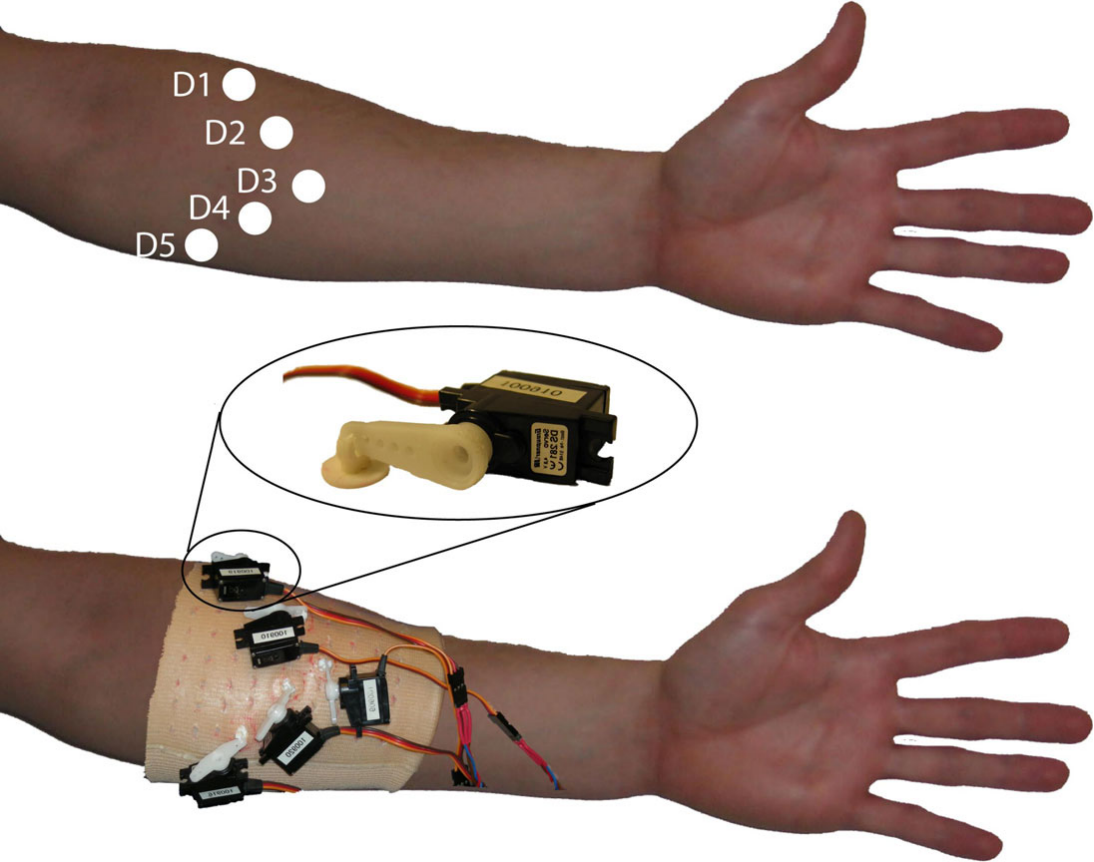
**Placement of actuators on the forearm reflecting the placements of the fingertips of an open hand drawn onto the forearm**.

The tactile display consists of the following components: a number of actuators to be placed on the amputee's residual limb providing tactile feedback and control electronics and software. A hardware interface is also available that makes it possible to control the tactile display from software running on computer in addition to the normal control which is based on the sensors placed on the prosthetic hand. This facilitates sensory feedback tests. The components and a prosthetic hand would be connected as described in the system block diagram presented in Fig. 1. The actuators used were digital servos (Graupner DS281, Germany). Affixed to the servo shaft is a 15 mm long lever at the end of which there is a plastic button, with a 12 mm diameter, see Fig. 2, that is pressed against the skin. The plastic button is fixed to the lever using a hinge mechanism to allow the plastic button to always be parallel to the skin. As the motor rotates the plastic button will cause a displacement of the skin. A microcontroller (MSP430F149, Texas Instruments) and associated hardware was used to control the digital servos.

**Figure 3:**
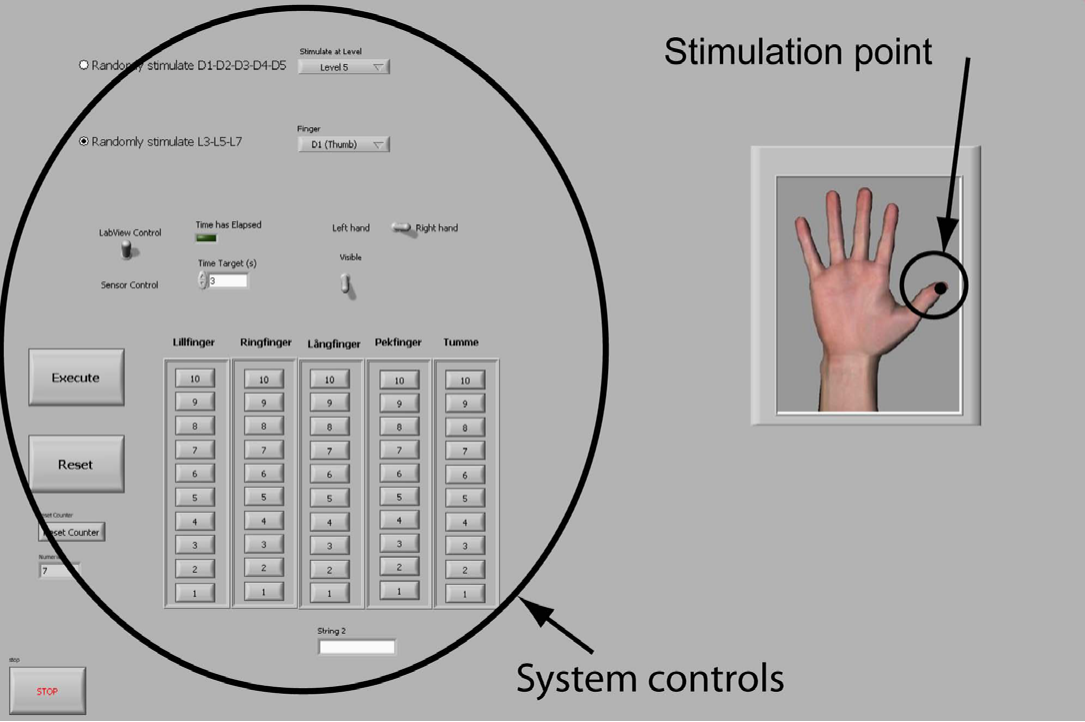
**Tactile Display LabView application frontend**. Controls and virtual hand as seen on the program used to generate the stimuli.

A user application for a computer was developed to enable a graphical user interface when testing the sensory feedback system (see Fig. 3). It communicates with the hardware through a virtual serial port on the computer (USB). The software is more flexible, easier to use and it is faster to develop new test programs and schemes compared to re-programming the microcontroller. During tests, the computer software was used to log the participant's performance, for randomly selecting stimuli and to provide feedback to participants during the training sessions. The software was built using LabVIEW to facilitate the test and use of the tactile display. The software was used to train the user by applying a stimuli and at the same time providing a visual representation of the stimuli. In the validation tests the program was used to generate randomly selected stimuli.

### Applicability of the system in an environment with myoelectric signals

EMG-signals, that would be used to control a prosthetic hand, would be influenced by the tactile display as the actuators for the tactile display and the EMG electrodes would be placed fairly close to one another. To see how this would influence the EMG, signal measurements with electrodes (Ag/AgCl Red Dot electrodes from 3 M) close to the actuators (~1 cm) were performed. The recorded EMG signal was subsequently filtered with FIR high-pass filter with a cut-off frequency of 20 Hz to remove the artefacts.

### Applied force and displacement of skin

**Figure 4:**
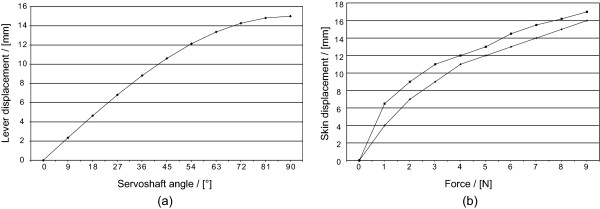
**Mapping between servo displacement and force**. An increasing servo displacement corresponds to an increase in force. This simplification was investigated by measuring force as a function of displacement of skin and tissue on two participants.

The actuators, i.e. servomotors, were operated by giving an angle command. A lever fixed on the axle of the servomotor was then pushing on the skin. As the servo arm can be rotated in fixed steps, the displacement of the button at the end of the lever can be calculated. The displacement of the button pushing on the skin then follows a sine function of the controlled motor angle (see Fig. 4a). An increasing displacement then corresponds to an increase in force. This simplification was investigated by measuring force as a function of displacement of skin and tissue on the forearm on two participants using a force gauge (Lutron FG-5000A, Lutron Electronics, Taiwan). The force gauge was mounted on a vertical "slider" with a ruler and the displacement was measured with this ruler while pressing the force gauge down on the skin to a certain force based on the reading from the force gauge.

### Evaluation on two healthy non-amputated subjects

To evaluate the system we performed experiments on two healthy non-amputees who have not previously been exposed to the system. The tactile display was placed (see Fig. 2) and fixated on the right forearm of the participant using an elastic restraining bandage. During the tests the participants were sitting in front of the computer screen with the arm in a relaxed position resting on a table. Earmuffs were worn at all times to prevent auditory stimuli from the sound of the servo motors. In each test the stimulus was randomly selected and presented for three seconds. The participant was blindfolded and had to guess finger discrimination and force level without any feedback during the test session.

Three different tests were carried out to investigate localization discrimination (i.e. which finger/site was being stimulated) of the fingers and force sensitivity at a single site of feedback. The first test was to discriminate between the thumb (D1), long finger (D3) and little finger (D5). The second test was to discriminate between all the fingers; thumb, index, long, ring and little finger (D1-D5, respectively) and the third was to test the force sensitivity on a single digit. A static mapping between skin displacement and force was used. Five levels, named L1-L3-L5-L7-L9, were used for the force sensitivity test. L9 representing the largest force. Each of the tests was proceeded by a learning phase consisting of an equal amount of stimulation as the actual test. The learning phase and the test phase lasted approximately ten minutes each. In the learning phase, the participants watched the computer screen showing the LabVIEW application which revealed the site of stimulation in the localization discrimination tests and the level of force in the force sensitivity tests. In this way, the participants learned the different stimulation sites and the force level.

## Results

A tactile display consisting of five actuators, control electronics and a test application has been designed and realised. The tactile display was controlled from a computer using a serial interface. A PC application with the capacity to generate arbitrary stimuli, selecting one or several actuators, individual levels on each actuator and duration of stimuli was used.

The relationship between force, skin displacement and angle value for the digital servos was studied. A calculated mapping between the different angles of the servomotor and the displacement of the lever can be seen in Fig. 4a. Measurements on skin indentation and force applied to the skin can be seen in Fig. 4b. As can be seen in Fig. 4 a mapping between force on the skin and angle command to the servo can be estimated.

**Figure 5:**
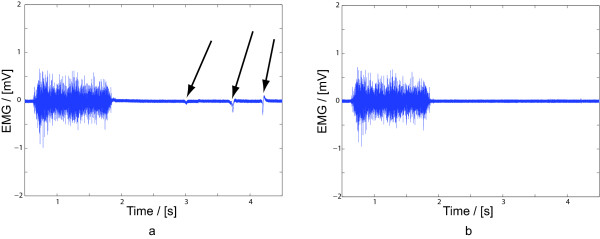
**Tactile Display influence on EMG-signal**. (a)Tactile Display influence on EMG-signal, shown by the arrows (b) Tactile Display artefacts removed from the EMG-signal with a high-pass filter.

An investigation was made to see if the displacement caused by the actuators of tactile display would influence EMG-signals. The recorded EMG signals during operation of the sensory feedback system can be seen in Fig. 5a. The arrows in the figure indicate when the sensory feedback system actuator was activated. Thus the closely located actuator did influence the EMG signal. The EMG signals were then filtered using a high-pass filter, to remove the artefacts caused by the tactile display. The filtered EMG signals can be seen in Fig. 5b were the artefacts have been completely removed.

A test of the tactile display was also carried out on healthy test subjects. Table 1 describes which test, the number of stimuli of a particular test, the results of the test and a nearest neighbour analysis. D1 to D5 denotes which digit the stimuli represents (D1 being the thumb and D5 being the little finger). L1 to L9 represents the force level of a stimuli. The percentage values indicate how many correct answers were given during the test and the number in parentheses the number of stimuli.

**Table 1:** Results from pilot test

	D1-D3-D5		D1-D2-D3-D4-D5		L1-L3-L5-L7-L9	
Participant	All	+/-	All	+/-	All	+/-

I	72% (25)	100%	48% (25)	96%	50% (25)	100%

II	100% (25)	100%	81% (55)	100%	66% (100)	100%

Three digit discrimination (D1-D3-D5), five digit discrimination (D1-D2-D3-D4-D5) and five level discrimination (L1-L3-L5-L7-L9). All means the actual result, +/- means that both positive and negative nearest neighbour are included.

The "+/-" columns shows that both a positive and a negative nearest neighbour answer is also deemed correct. E. g. an answer stating digit 4 or digit 2 would also be judged as correct for a stimulus at digit 3. Using an analysis such as the one described above, it is possible to see a bias in the answers. Thus, when the answer was incorrect it was most often one of the neighbouring digits.

## Discussion and conclusions

We have previously presented a system for sensory feedback in hand prostheses to provide the user with a sense of touch and here we elaborate the details of the technical solution. The system aims at relocating tactile input from a hand prosthesis equipped with sensors to the forearm skin using actuators mounted on the forearm, thus providing sensory information to the user. The concept utilises the neural mechanoreceptors for pressure in the forearm skin. Every time the user touches and manipulates an object with the prosthesis, the mechanoreceptors of the forearm skin are activated by the tactile display.

One concern was that the actuator relaying pressure onto the forearm would generate artefacts in the recorded myoelectric signals as electrodes would also be located on the forearm. The EMG measurement from very closely located electrodes (1 cm) shows some influence on the recorded EMG that was easily filtered out by a high pass filter with a 20 Hz corner frequency. This filter would also reduce artefacts in the EMG originating from movement of the prosthesis. Filtering out signals below 20 Hz will also filter out EMG signals. However, the lower frequencies have been filtered out in earlier works of myoelectric control [25] where a high pass filter with a corner frequency as high as 200 Hz was reported with a high recognition ratio on a group of amputees. A cut-off frequency of 20 Hz should therefore not reduce the performance of a myoelectric control system.

An important issue is that the spatial resolution of the forearm is an order of magnitude less than the spatial resolution of the fingertips. In the work of Weinstein [26], two-point discrimination on different parts of the body have been investigated

The two-point discrimination of the forearm is about four cm suggesting this to be interdistance between actuators which closely corresponds with our placement. Thus the actuator elements need to be placed with a quite high degree of separation on the forearm. This put restrains on how well the sensory input could be fed back to the participants. The actuators were initially placed on a line except for the actuator for the thumb that was placed towards the hand. Testing different placements later revealed that a placement of the actuators reflecting the placements of the fingertips of an open hand drawn onto the forearm was more intuitive and also increased the distance between the actuators.

The proposed sensory feedback system provides dynamic and static pressure feedback to the user. This could also be used on patients that have undergone targeted muscle reinnervation, placing the tactile display on the reinnervated areas of the chest. However, this type of procedure is more suitable for a shoulder disarticulation amputation as to the invasive nature of the procedure.

The accuracy presented in our results with two individuals shows promising outcomes for the use of such a tactile display with five actuators to represent the five fingers. It is likely that an even higher accuracy would be gained after more training. Different methods of training could also help, e.g. reinforced learning were the participant is blindfolded and guessing which stimuli that have been applied where after the test supervisor provides feedback in the form of a correct answer. Having more sensing elements on the thenar and hypothenar regions of the hand seems to be a natural region to cover and may increase haptic perception. However, the result also shows that the five fingers were not easily separated by all participants and increasing the number of actuators would definitely demand more training of the user. The number of sensors and hence the number of actuators were based on having one sensing element per finger of the human hand.

The force level discrimination experiment had basically the same resolution as the finger discrimination experiment. Usually the force delivered by the actuator would be measured and controlled, however, using the displacement of the actuator lever will give a good force estimate.

A disadvantage with servomotors is their power consumption which is quite high. Power consumption will always be an issue in prosthetics and the usefulness of the proposed device as perceived by the user vs. power consumption are also fundamental parameters that needs to be investigated.

Actuators were mounted on the forearm on two participants providing sensory feedback addressing both spatial as well as the level of force of the stimuli. The evaluation of the system in the present study as well in the previously presented study where the system was applied on 11 healthy test subjects [21] suggests that this is a viable method for providing sensory feedback to forearm amputees using prosthetic devices.

A quite distal placement of actuators of the same size as the ones here proposed should be possible in a prosthetic socket without compromising the appearance of the socket. If the actuators were to be integrated into a socket, the socket itself would provide some attenuation of the sound generated by the motors. Using a sensory feedback system in a prosthetic device would also be to improve user acceptance of the prosthetic device as a whole.

Although it remains unrealistic to expect the proposed sensory feedback system to provide as accurate sensibility as a real hand, our system does provide a relatively simple and non-invasive way to restore rudimentary sensory feedback in prostheses used by transradial/-humeral amputees. Future work will focus on application of the system on amputees, training methods, cognitive aspects and finding a solution with actuators that have a lower power consumption.

## Competing interests

The authors declare that they have no competing interests.

## Authors' contributions

CA constructed the tactile display and the LabView application, conducted the experiments and drafted the manuscript. CB, GL, BR, FS participated in the technical design, the planning of the study and helped draft the manuscript. All authors have read and approved this manuscript.
